# Exploring Parenting Profiles to Understand Who Benefits from the Incredible Years Parenting Program

**DOI:** 10.1007/s11121-022-01364-6

**Published:** 2022-03-19

**Authors:** Joyce Weeland, Patty Leijten, Bram Orobio de Castro, Ankie Menting, Geertjan Overbeek, Maartje Raaijmakers, Joran Jongerling, Walter Matthys

**Affiliations:** 1 Erasmus School of Social and Behavioural Sciences, Erasmus University Rotterdam, P.O. Box 1738, 3000 DR Rotterdam, The Netherlands; 2 Research Institute Child Development and Education, University of Amsterdam, Amsterdam, The Netherlands; 3 Developmental Psychology, Utrecht University, Utrecht, The Netherlands; 4 De Opvoedpoli, Utrecht, The Netherlands; 5 Tilburg School of Social and Behavioral Sciences, Department of Methodology, Tilburg University, Tilburg, The Netherlands; 6 Department of Clinical Child and Family Studies, Utrecht University, Utrecht, The Netherlands

**Keywords:** Disruptive child behavior, Incredible Years, Moderation, Parenting program, Parenting profiles

## Abstract

Behavioral parenting programs are a theory-driven and evidence-based approach for reducing disruptive child behavior. Although these programs are effective on average, they are not equally effective in all families. Decades of moderation research has yielded very few consistent moderators, and we therefore still have little knowledge of who benefits from these programs and little understanding why some families benefit more than others. This study applied a baseline target moderation model to a parenting program, by (1) identifying parenting profiles at baseline, (2) exploring their correlations with other family characteristics and their stability, and (3) assessing whether they moderate intervention effects on child behavior. Individual participant data from four Dutch studies on the Incredible Years (IY) parenting program were used (*N* = 785 caregiver–child dyads). Children (58.2% boys) were at risk of disruptive behavior problems and aged between 2 and 11 years of age (*M* = 5.85 years; *SD* = 1.59). Latent profile analyses indicated three distinct baseline parenting profiles, which we labeled as follows: *Low Involvement* (81.4%), *High Involvement* (8.4%), and *Harsh Parenting* (10.1%). The profiles caregivers were allocated to were associated with their education, minority status, being a single caregiver, and the severity of disruptive child behavior. We found neither evidence that baseline parenting profiles changed due to participation in IY nor evidence that the profiles predicted program effects on child behavior. Our findings do not support the baseline target moderation hypothesis but raise new questions on how parenting programs may work similarly or differently for different families.

**Supplementary Information:**

The online version contains supplementary material available at 10.1007/s11121-022-01364-6.

Parenting programs are an evidence-based intervention strategy for the prevention and treatment of disruptive behavior problems in children (Piquero et al., 2016). However, these programs benefit some families more than others (Pelham et al., 2017; Thijssen et al., 2017; van Aar et al., 2019). Some studies show that, even between families participating in the same program, a small number of families benefit greatly, while large numbers of families benefit very little (for example in the study of van Aar et al. (2019), Cohen’s *d* = 1.45 for 18% vs. *d* = 0.12 for 82% of the families). Many studies have tried to identify family characteristics that may help us predict who benefits more or less (i.e., moderation research,Gardner et al., 2010; Leijten et al., 2018; Weeland et al., 2017). However, very few consistent moderators have been identified, in both single studies (McMahon et al., 2021; Shelleby & Shaw, 2014) and individual participant meta-analyses (Leijten et al., 2018). Moreover, many of these moderators provide us with little insight in the mechanisms of change underlying differential effectiveness, as they do not assess the actual family dynamics these programs aim to change. For example, how do the severity of disruptive behaviors or caregiver depression explain why families benefit more or less from a parenting program (McMahon et al., 2021)? To date, we therefore still have little knowledge about why some families benefit more or less than others. The current study applies a different approach to moderation, based on the baseline target–mediated moderation model (BTMM) (Howe et al., 2016).

Parenting programs are hypothesized to reduce disruptive child behavior by changing how caregivers prepare for and react to child behavior (Forehand et al., 2014). If parenting programs indeed work through changes in these parenting behaviors, then these programs should be particularly suitable for families that experience more problems with these parenting behaviors before participation (i.e., at baseline). In general, parenting programs may thus be more effective in families in which parenting problems are more severe (i.e., less frequent positive parenting behaviors and more frequent negative parenting behaviors). In these families, there is more room for improvement in parenting and, in turn, a greater potential for impacting more distal intervention outcomes (in case of parenting programs: child behavior). Indeed, caregiver-reported critical, harsh, and ineffective parenting at baseline was found to predict intervention effects of a popular parenting program (Beauchaine et al., 2005), the Incredible Years (IY) (Webster-Stratton, 2008). Following the principles of the BTMM (Howe et al., 2016), we will assess whether how much families benefit from a parenting program for disruptive child behavior depends on their parenting behavior at baseline (i.e., baseline target moderation (BTM)).

Although this approach may seem intuitive, applying this to parenting interventions is complex, as parenting is a multi-dimensional construct incorporating both positive behaviors (e.g., monitoring and praise) and negative behaviors (e.g., corporal punishment and shouting). This complexity is lost when collapsing parenting behaviors in broad categories, such as “positive” versus “negative” parenting. Moreover, positive and negative parenting behaviors are not necessarily two sides of the same coin (Borden et al., 2014; Leijten et al., 2018) and caregivers may be more inclined to use some aspects of positive or negative parenting (e.g., praising children for positive behavior) than others (e.g., using tangible rewards for positive behavior). Different studies in different populations have tried to capture this complexity (e.g., Borden et al., 2014; Cook et al., 2012; Heberle et al., 2015). For example, in a sample of low-income mothers, three parenting profiles were found: “Developmental parenting” characterized by relatively high scores on most supportive parenting behaviors and by relatively low scores on emotionally negative behaviors, “Unsupportive parenting” characterized by relatively low scores on most supportive parenting behaviors and by moderate scores on emotionally negative behaviors, and “Negative parenting” characterized by relatively high scores on emotionally negative behaviors and by moderate scores on supportive behaviors (Cook et al., 2012).

The complex interplay between various aspects of positive and negative parenting behaviors may help explain differences in parenting program effectiveness. Above and beyond the severity of problems in the parenting domain, specific combinations of parenting behaviors used by caregivers before their participation in a parenting program may predict how much a family benefits from this program. This may be explained in different ways. First, different parenting behaviors may interact. For example, the effectiveness of positive parenting behaviors such as praise may depend on how they are delivered (e.g., more effective when delivered with warmth and enthusiasm). Therefore, these behaviors may not be effective in decreasing disruptive child behavior if combined with harsh parenting (e.g., shouting or corporal punishment) (Dadds & Hawes, 2006). Parenting programs may thus also be effective for families in which caregivers already frequently use positive parenting behaviors if they also frequently use harsh parenting behaviors at baseline.

Second, specific combinations of parenting behaviors may reflect underlying problems, which may or may not be successfully addressed in the program. For example, low involvement (characterized by low monitoring and low expressed affect including praise) may reflect stress or low self-efficacy in caregivers (Jones & Prinz, 2005). Harsh parenting (characterized by negative emotional expressions including shouting and physical punishment) may reflect anger and irritability due to negative attributions about disruptive child behavior (Beckerman et al., 2018). Such underlying problems may, in some cases, prevent changes in parenting behavior during participation in a parenting program.

Third, certain child characteristics may evoke or intensify the use of specific parenting strategies. For example, behaviors related to childhood attention-deficit hyperactivity disorder (ADHD) may evoke coercive parenting (characterized by shouting, threatening, but also laxness) and, through this, indirectly lead to and maintain disruptive behavior (Beauchaine et al., 2010). Such underlying problems may prevent program-induced changes in parenting behavior from impacting child behavior. Families with these underlying problems may therefore not benefit from a parenting program alone and may need additional or different forms of support to reduce disruptive child behavior. Both the intensity and the nature of problems in parenting possibly underlie differential intervention effects. Traditional single-variable moderation analyses mask such meaningful interaction effects.

## The Present Study

The first aim of the current study is to explore how caregivers of children at risk of, or who already developed symptoms of, disruptive behavior problems are different and similar to each other in their parenting. We use latent profile analyses to (1) identify subgroups of families based on their similarities on a *constellation* of parenting behaviors that are specifically targeted in the intervention and explore how stable these constellations, or profiles, are over time and (2) how they relate to other family characteristics (e.g., caregivers’ educational background and severity of children’s disruptive behavior). We specifically focus on parenting behaviors, rather than cognitions (e.g., self-efficacy) or mental health (e.g., depression), because in line with BTM principles, we focus on pre-intervention characteristics that are directly targeted in the intervention. The main aim of this study is to assess whether parenting profiles predict how much families benefit from the parenting program IY in terms of reduced disruptive child behavior. Using pre-intervention profiles as a moderator allows us to consider how multiple putative moderators cluster together and affect intervention effectiveness in one model. Because most studies are only powered to test main effects and/or single moderator effects, we tested whether parenting profiles predict intervention effects in pooled data from four studies. To create a larger and more diverse sample (e.g., more variance in constructs of interest), we combined individual family, item-level data of four studies on the effectiveness of the evidence-based IY program (Webster-Stratton, 2008) in the Netherlands. This may increase power, sample diversity, and help us to also identify less prevalent constellations of parenting behaviors.

## Methods

### Procedure

Data were obtained from four studies on the effectiveness of the IY parenting program (these data have been previously used to assess single moderators; see Leijten et al., 2018). Two studies (#3 and #4) were randomized controlled trials; one was a matched control group study (#1), and one study combined random allocation and full allocation to the intervention group (#2). Two studies (#1 and #4) were conducted in an indicated prevention setting (following screening for heightened, borderline, or clinical levels of disruptive child behavior): one study (#2) was in a selective prevention setting, and one study (#3) was a mix of selective prevention and treatment. In three studies (#1, #2, and #4), participants in the control condition did not receive IY but were free to seek alternative assistance or treatment; in one study (#3), participants in the control condition were on a waiting list to receive IY. Participants from all studies signed informed consent, and study protocols were approved by Internal (Medical) Ethical Review Boards (see Leijten et al., 2017; Menting et al., 2014; Posthumus et al., 2012; Weeland et al., 2017). See Online Resource 1 for information on study characteristics and participant flow diagrams per study.

Across the four studies, about half of caregiver–child dyads (57.5%) were allocated to the intervention group and were offered the IY program. In none of the studies, significant differences between families allocated in the control or intervention groups on disruptive child behavior, parenting behaviors, or demographics (parental and child age, minority background, education, and having a partner) were found: in study #1, *F*(13, 120) = 1.67, *p* = 0.08, and Wilks’ Λ = 0.85; in study #2, *F*(13, 69) = 0.80, *p* = 0.66, and Wilks’ Λ = 0.150; in study #3, *F*(13, 85) = 0.98 and *p* = 0.48; and in study #4, *F*(13, 365) = 0.86, *p* = 0.60, and Wilks’ Λ = 0.03.

### Participants

Participants were 785 Dutch caregiver–child dyads. Children (58.2% boys) were between 2 and 11 years of age (*M* = 5.85 years; SD = 1.59). Most children (74.0%) scored in the top 25% of disruptive behaviors in the Dutch population and about a quarter (27.7%) in the top 5% (based on age- and sex-specific Dutch norm scores; see Weeland et al., 2018). Caregivers, mostly mothers (93.6%), were between 20 and 53 years of age (*M* = 36.17; SD = 5.64). One in seven (14.1%) reported being a single caregiver, a third (35.0%) being low educated (i.e., only primary or secondary education), and a third (30.1%) having a minority background (i.e., non-white). Families participated in pre-intervention (i.e., baseline) and post-intervention (i.e., directly after the intervention) assessments of parenting and child behavior.

### Measures

#### Disruptive Behavior

In all included studies, the Eyberg Child Behavior Inventory (ECBI) (Eyberg & Pincus, 1999) was used to assess parent-reported disruptive child behavior. We used the intensity scale, consisting of 36 items measuring the frequency of disruptive behavior (e.g., *Acts defiant when told to do something*) on a 7-point scale (1 = *never* to 7 = *always*). The ECBI showed good reliability and validity in Dutch samples (Abrahamse et al., 2015), and the internal consistency of the intensity scale in the current study was also good (*α* = 0.96 and McDonald’s *ω* = 0.90 at baseline).

#### Parenting

Parenting behavior was assessed using the Parenting Practices Inventory (PPI) (Webster-Stratton, 2001) (in studies #1, #3, and #4) and the Alabama Parenting Questionnaire (APQ) (Essau et al., 2006) (in study #2). Both instruments are well validated to assess parenting behaviors (Dadds et al., 2003; Leijten et al., 2018). Seven parenting behaviors, specifically targeted in IY, were selected and defined: corporal punishment, threatening, laxness, shouting, praise, tangible rewards, and monitoring. Following the procedure by Leijten et al. (2018), data from the PPI and APQ were integrated by selecting from each instrument the items that fit the seven parenting behaviors (Online Resource 2). For example, corporal punishment was defined as any physical punishment such as slapping or spanking. This was reflected in 3 APQ items (e.g., *You slap your child when he/she has done something wrong*) and 6 PPI items (e.g., *How often do you do each of the following things when your child misbehave: Slap or hit your child*?). The scales using APQ items were recoded to fit a 7-point scale (1 = 1, 2 = 2.5, 3 = 4, 4 = 5.5, 5 = 7). Internal consistency was acceptable to good for all scales at baseline (McDonald’s *ω* ≥ 0.60, Table 2.1 Online Resource 2).

### Parenting Program: the Incredible Years Program

The IY program is a group behavioral parent training program consisting of 12–18 weekly sessions, depending on the version of the program (Webster-Stratton, 2008). The program starts with a focus on positive parenting behaviors such as praise before discussing effective and consistent limit setting, ignoring unwanted behavior, and finally, time-out strategies. During the sessions, caregivers watch video vignettes of caregivers and children interacting (Dutch subtitles were used in the vignettes), act in role-plays, have brainstorming sessions, and exchange experiences and ideas in small groups. The program uses a collaborative setting: group leaders establish themselves as facilitators rather than as experts. Group leaders encourage caregivers to solve problems and to help one another solve problems to ensure maintenance of the intervention effects. In each group, at least one of the two group leaders was a certified IY group leader. Of the caregivers allocated to the intervention, 88.0% participated in IY (i.e., attended at least one session). On average, these participants attended 73.0% of the IY sessions offered to them.

### Analyses

Data were analyzed using MPlus (Muthén & Muthén, 2017). Our research question was answered in three steps (Fig. 5.1; see Online Resource 5 for an illustration of the baseline target moderation model we assessed). In step 1, families were allocated to parenting profiles using latent profile analyses (i.e., a latent class analysis with continuous data). Profiles were based on baseline data of seven parenting scales: praise, tangible rewards, monitoring, shouting, threatening, corporal punishment, and laxness. Profile solutions with one to six latent profiles were ran sequentially and were evaluated based on the following: (a) three fit indices (Bayesian information criterion (BIC), Akaike information criterion (AIC), and the Lo–Mendell–Rubin-adjusted likelihood ratio test (LR test), (b) entropy (i.e., estimate of the probability that each participant is in each of the classes) and mean class probabilities, (c) profile size (i.e., % of participants in each profile), and (d) theoretical plausibility. Complete data on parenting behaviors, needed to allocate families to a profile, were available for 747 families at baseline. After the selection of the best fitting profile solution, profile membership for each family was exported and used as a predictor of the study of origin, condition, retention, disruptive child behavior, family characteristics, and finally, intervention effectiveness. To explore whether parenting profiles were stable or changed over time and whether change was predicted by the intervention, we repeated the procedure of step 1 using data on the seven parenting scales post-intervention in step 2. Complete data, needed to allocate families to a profile, were available for 687 families post-intervention.

In step 3, we assessed—possible differential—effects of condition (IY vs. control) on disruptive child behavior post-intervention (controlling for disruptive child behavior at baseline) using a path analysis. In these models, missing data were treated using full information maximum likelihood (FIML). Missing data on the child behavior post-intervention (i.e., the intervention outcome) was related to caregiver education, minority status, and age, to child sex and to the original study families originated from. These variables were therefore included in the models as covariates to optimize the FIML procedure. Model fit was assessed using the chi-square, root mean square error of approximation (RMSEA) (model fit satisfactory when the value was < 0.08), and confirmatory factor index (CFI) (model fit satisfactory when the value was > 0.90) (Hu & Bentler, 1999). Because data were skewed (for distribution of baseline data, see Figs. 3.1–3.8 in Online Resource 3), we report maximum likelihood robust (MLR) fit indices. We used profile membership as a grouping variable to assess whether the path from condition (IY vs. control) to disruptive child behavior post-intervention differed between profiles. We compared a path model in which this path was estimated for each profile to a path model in which this path was constrained to be equal across profiles. Chi-square difference testing using the Satorra–Bentler-scaled chi-square was used to compare the model fit between these nested models. In case of better fit in the unconstrained model, path coefficients were compared between profiles using 5000 bootstrapped 95% confidence intervals.

## Results

### Baseline Parenting Profiles

The sequential profile analyses with baseline data showed that AIC and BIC decreased when the number of profiles increased, indicating increased model fit with more profiles. The Lo–Mendell–Rubin-adjusted LRT was significant up to five profiles, meaning that the increase in model fit was no longer significant when we went from four to five profiles. Entropy was above 0.90 for the two- and three-profile solutions (indicating good classification quality), but under 0.80 for the four- and five-profile solutions. Class probabilities were above 0.92 and 0.88 for all classes for the two- and three-profile solutions, respectively, but fell under 0.71 in the four- and five-profile solutions (Table 1). Because of the good fit with the data, the high classification quality, and the theoretical plausibility of the profiles, a 3-profile solution was selected (entropy = 0.92, class probabilities > 0.88). We first tested whether the profiles differed in study characteristics (study of origin, condition, and retention [i.e., whether post-intervention data was available]). Profile membership predicted the study of origin (however, all profiles contained families from each of the four studies; Table 2) and percentage families allocated to the intervention group (however, all profiles contained > 50% of families allocated to the intervention; Table 2), but not retention (*χ*^2^ = 0.00, *df* = 0, *p* < 0.001; RMSEA < 0.001; CFI = 1.00; SRMR < 0.001 [saturated model]; condition: *B* = 0.07, SE = 0.03, *p* = 0.02; study of origin: *B* =  −0.32, SE = 0.07, *p* < 0.001; retention: *B* = 0.03, SE = 0.02, *p* = 0.09). We controlled for the study from which families originate in further analyses.

**Table 1:** Results of the profile analyses

# profiles	AIC	BIC	Lo–Mendell–Rubin-adjusted LR test		Entropy	Lowest class probability
1	16,285.349	16,349.974	–	–	–	–
2	15,881.605	15,983.158	411.961	*p* < 0.001	.947	.928
3	15,667.270	15,805.752	226.064	*p* < 0.001	.924	.875
4	15,565.190	15,740.601	115.890	*p* = 0.008	.782	.654
5	15,483.718	15,696.057	73.740	*p* = 0.089	.769	.676
6	15,395.345	15,644.613	87.716	*p* = 0.288	.793	.705

**Table 2:** Descriptives for and differences between the three baseline parenting profiles

	**All (*N* = 747)**		**Profile 1 (*n* = 608)**			**Profile 2 (*n* = 63)**			**Profile 3 (*n* = 76)**		
	***M***	**SE**	***M***	**SE**	**95% CI**	***M***	**SE**	**95% CI**	***M***	**SE**	**95% CI**
Corporal punishment T1*	1.54	0.03	1.32	0.02	1.29–1.35	1.54	0.07	1.41–1.69	3.33	0.08	3.12–3.49
Shouting T1*	3.53	0.05	3.48	0.05	3.39–3.56	2.89	0.19	2.52–3.26	4.53	0.13	4.26–4.78
Praise T1	5.00	0.04	4.93	0.04	4.85–5.02	5.87	0.13	5.60–6.12	4.78	0.13	4.52–5.04
Tangible rewards T1*	3.00	0.05	2.64	0.04	2.57–2.70	6.11	0.10	5.92–6.31	3.31	0.15	3.02–3.61
Monitoring T1*	5.76	0.04	5.70	0.43	5.61–5.78	6.57	0.09	6.38–6.73	5.61	0.14	5.33–5.86
Threatening T1*	3.30	0.06	3.20	0.60	3.08–3.32	3.68	0.22	3.27–4.11	3.81	0.19	3.45–4.20
Laxness T1*	2.30	0.04	2.84	0.44	2.75–2.92	3.21	0.22	2.79–3.64	3.43	0.17	3.09–3.74
Disruptive child behavior T1*	127.35	0.96	129.57	1.01	127.56–131.61	106.30	4.07	98.44–114.48	126.86	3.08	121.70–132.89
	**All (*N* = 747)**		**Profile 1 (*n* = 608)**			**Profile 2 (*n* = 63)**			**Profile 3 (*n* = 76)**		
**Study characteristics (%)**											
Intervention group*	57.60		56.30			69.80			67.10		
Study 1*	18.00		20.20			1.60			22.40		
Study 2	12.90		4.40			74.60			23.70		
Study 3	19.90		17.40			14.30			18.40		
Study 4	49.30		57.90			9.50			35.50		
Retention	87.90		90.50			71.40			89.50		
**Family characteristics (%)**											
Child disruptive behavior > 90^th^ percentile ^a^	27.70		18.70			7.90			29.90		
Single caregiver*	14.10		11.10			41.30			17.30		
Low educated*	35.10		27.90			70.20			47.90		
Minority status*	30.10		21.80			67.8			42.70		
IY sessions attended (% of offered)^b^	63.41		64.70			58.05			60.46		

^a^Norm scores based on age- and sex-specific Dutch norm scores (Weeland et al., 2018)

^b^Participants allocated to in the intervention condition, including participants who did not attend any sessions

^*^Significant difference between profiles: corporal punishment (*B* = 0.88, SE = 0.04, *p* < 0.001), shouting (*B* = 0.41, SE = 0.07, *p* < 0.001), praise (*B* = 0.03, SE = 0.07, *p* = 0.71), tangible rewards (*B* = 0.76, SE = 0.10, *p* < 0.001), monitoring (*B* = 0.23, SE = 0.06, *p* < 0.001), threatening (*B* = 0.39, SE = 0.09, *p* < 0.001), laxness (*B* = 0.32, SE = 0.08, *p* < 0.001), education (*B* =  −0.15, SE = 0.03, *p* < 0.001), minority status (*B* = 0.16, SE = 0.03, *p* < 0.001), single caregiver (*B* = 0.06, SE = 0.02, *p* < 0.001), disruptive behavior: sum scores (*B* =  −3.66, SE = 1.63, *p* = 0.03), disruptive behavior: norm scores (*B* =  −0.10, SE = 0.08, *p* = 0.22), sessions attended (*B* =  −4.22, SE = 2.48, *p* = 0.09)

**Fig. 1:**
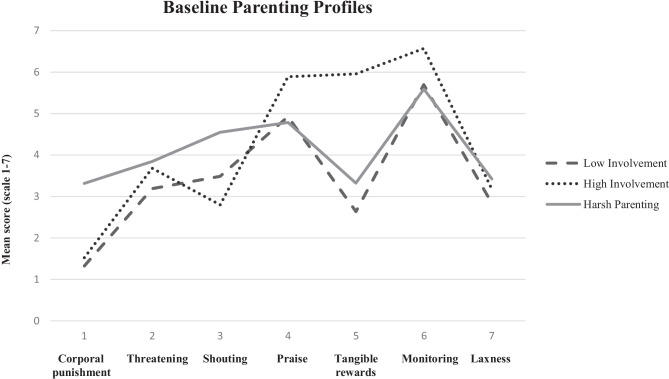
Parenting profiles pre-intervention (i.e., baseline)

The three parenting profiles reflect how frequently caregivers report the use of specific parenting behaviors, relative to caregivers in the other profiles. Controlling for the study families originated from, profile membership significantly predicted the reported use of all parenting behaviors, except for praise (*χ*^2^ = 0.00, *df* = 0, *p* < 0.001; RMSEA < 0.001; CFI = 1.00; SRMR < 0.001 [saturated model], Table 2). The use of corporal punishment, tangible rewards, and monitoring seemingly discriminated most between profiles (Fig. 1). Caregivers allocated to profile 1 (*n* = 608 [81.4%], average class probability = 0.983) reported relatively low frequencies of most positive and negative parenting behaviors compared to the other profiles, except for shouting (which was in between the other two profiles; Fig. 1). These caregivers reported significantly less use of corporal punishment and tangible rewards than both other profiles, less monitoring compared to profile 2, and less threatening and laxness compared to profile 3 (95% CIs in Table 2). Compared to the full sample, the mean of all reported parenting behaviors was below the sample mean but deviated less than half a standard deviation. We label this profile Low Involvement, reflecting relatively limited use of both positive and negative parenting behaviors.

Caregivers allocated to profile 2 (*n* = 63 [8.4%], average class probability = 0.875) report relatively frequent use of positive parenting behaviors. The use of corporal punishment, threatening, and laxness was in between the other two profiles (Fig. 1). These caregivers reported significantly more monitoring and tangible rewards and less shouting than caregivers in both other profiles (95% CIs in Table 2). Compared to the full sample, these caregivers reported relatively frequent use of all parenting behaviors (except for corporal punishment and shouting) and, specifically, frequent use of tangible rewards, monitoring, and laxness (2.32, 0.76, and 0.59 SD, respectively, above the sample mean). We label this profile High Involvement, reflecting relatively frequent use of positive behaviors and moderate to frequent use of negative behaviors. Caregivers allocated to profile 3 (*n* = 76 [10.1%], average class probability = 0.936) report frequent use of all negative parenting behaviors compared to other profiles. They also reported the lowest levels of praise, and tangible rewards and monitoring were between the other two profiles (Fig. 1). Indeed, these caregivers reported significantly more use of corporal punishment and shouting than caregivers in both other profiles (95% CIs in Table 2). Compared to the full sample, these caregivers reported relatively frequent use of corporal punishment, shouting, and laxness (2.38, 0.82, and 0.74 SD, respectively, above the sample mean). We label this profile Harsh Parenting, reflecting relatively frequent use of harsh parenting behaviors such as corporal punishment and threatening and low-to-moderate use of positive parenting behaviors.

We explored whether families in different profiles differed on family (education, single-caregiver household, minority status, number of IY sessions attended) and child (disruptive behavior severity) characteristics. Profile membership significantly predicted caregivers’ education level, minority status, being a single-caregiver household, and the level of children’s disruptive behavior at baseline, but not the percentage of children scoring above the 90^th^ percentile or the number of IY sessions caregivers attended (*χ*^2^ = 0.00, *df* = 0, *p* < 0.001; RMSEA < 0.001; CFI = 1.00; SRMR < 0.001 [saturated model]; see note of Table 2 for results and Table 2 for descriptive variables). Families in the *Low Involvement profile* were mostly majority (i.e., white), with higher education, and two-caregiver households, with children scoring relatively high on disruptive behavior problems: almost a third (27.7%) scored above the 90^th^ percentile. Families in the High Involvement profile were mostly minority, with lower education, and single-caregiver households. Children scored relatively low on disruptive child behavior: two thirds (64.0%) scored below the 70^th^ percentile. Families in the Harsh Parenting profile seemed more diverse, and about half of these caregivers were of minority background (42.0%) and have lower education (49.7%); the other half was not. About a third (29.3%) scored above the 90^th^ percentile, while about a third scored much lower (below the 70^th^ percentile).

### Stability of Parenting Profiles

**Fig. 2:**
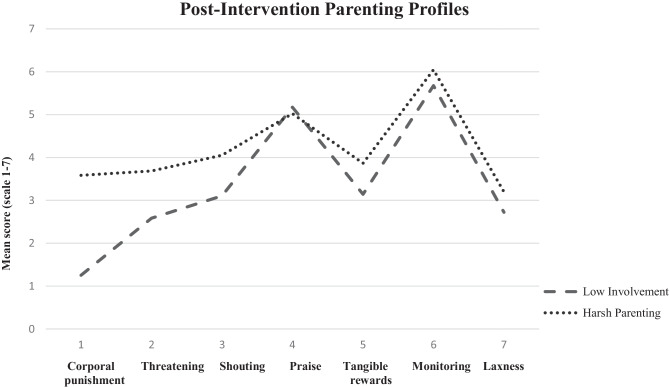
Parenting profiles post-intervention

The sequential profile analyses with post-intervention data lead to a 2-profile solution (entropy = 0.975, class probabilities > 0.940, Table 4.1, Online Resource 4). The two parenting profiles significantly differed on all parenting behaviors, except praise and monitoring, on family characteristics, but not on disruptive child behavior (Table 4.2, Online Resource 4). Post-intervention profile 1 (*n* = 644, average class probability = 0.998) closely resembles the baseline profile “Low Involvement.” Post-intervention profile 2 (*n* = 46, average class probability = 0.950) closely resembles the baseline profile “High Involvement” (Fig. 2). The clustering of parenting behaviors thus seemed relatively consistent over time.

Assuming post-intervention profiles 1 and 2 are indeed comparable to the baseline Low Involvement and Harsh Parenting profiles, respectively, we tested profile stability by assessing change in profile membership between pre- and post-intervention. Baseline profile membership significantly predicted change in profile membership. The baseline Low Involvement seemed relatively stable over time (2.7% of caregivers in this profile changed profile membership), whereas the baseline High Involvement and Harsh Parenting seemed less stable over time. The relative low retention rate in the Harsh Parenting profile (i.e., no post-test data available for 28.6% vs. 9.5% and 10.5% for caregivers in the other profiles) may have contributed to the disappearance of this profile. Condition (IY vs. control) or an interaction between condition and baseline profile did not predict profile change (Online Resource 4).

### Differential Effectiveness of the Intervention: Baseline Target Moderation

A path model in which condition (IY vs. intervention) predicted disruptive behavior post-intervention, controlling for disruptive child behavior baseline, study from which families originated, child age, caregiver sex, education, minority status, and being a single caregiver), was estimated (for a path diagram, see Fig. 5.2, Online Resource 5). The model had excellent fit (*χ*^2^ = 3.952, *df* = 5, *p* = 0.556; RMSEA < 0.001; CFI = 1.00; SRMR = 0.013). These results indicate that condition did not have a significant effect on disruptive child behavior at baseline but did have a significant effect on disruptive child behavior post-intervention. Caregivers in the intervention group reported significantly less child disruptive behavior (*B* =  −0.228, SE = 0.037, *p* < 0.001; Cohen’s *d* =  −0.27) at post-test compared to caregivers in the control group (means reported in Table 6.1, Online Resource 6). Chi-square value decreased when the path from condition to disruptive behavior post-intervention was estimated freely (*χ*^2^ = 3.188), compared to when this path was constrained to be equal across profiles (*χ*^2^ = 3.952). This indicates better model fit in the freely estimated model. However, this difference was not significant (difference =  −0.806, *p* = 1.00). We therefore found no evidence that the effect of IY on disruptive child behavior was different across profiles (for effects per profile, see Table 6.2, Online Resource 6).

## Additional Analyses

As an additional test of the validity of using parenting profiles over individual parenting behaviors as moderators of the intervention effect, we also explored whether individual parenting behaviors baseline moderated intervention effects on disruptive child behavior post-intervention. We used a path model including paths from condition, individual parenting behaviors, and their interaction on disruptive child behavior, again controlling for disruptive child behavior at baseline, the study from which families originated, child age, caregiver sex, education, minority status, and being a single caregiver. We found no evidence of a differential effect of IY on disruptive behavior based on individual baseline parenting behaviors (Online Resource 6, Table 6.3 and Fig. 6.1).

## Conclusion and Discussion

Parenting programs such as IY have been designed to prevent or decrease disruptive behavior problems in young children via changes in parenting behavior. Although generally effective, they do not benefit all families (e.g., Pelham et al., 2017; van Aar et al., 2019). Based on the theory of change of parenting programs, one would expect that how much families benefit depends on the nature of baseline parenting behavior: benefits should primarily be obtained by families with more severe parenting problems, or problems specifically targeted in the intervention. This study applied a baseline target moderation (BTM) model to the parenting program IY and aimed to assess (1) similarities and differences in parenting behaviors among caregivers enrolled in studies of the IY parenting program using latent profile analyses; (2) associations between parenting profiles, family characteristics, and profile stability; and (3) whether the identified baseline parenting profiles predicted how much families benefitted from the program. We identified three distinct parenting profiles which were related to caregiver education, minority status, being a single caregiver, as well as to child disruptive behavior. Stability of the profiles over time differed by profile but seemed not affected by IY. Importantly, IY proved effective in reducing disruptive child behavior, but the size of this effect was independent of the baseline parenting profiles. We thus found no evidence for the baseline target moderation model.

Our parenting profiles were thus not able to explain the previously found heterogeneity in the effectiveness of IY. They do give us new insights in differences in parenting behavior between families and raise important issues for future research. First, the identified baseline parenting profiles were characterized by differences in most parenting behaviors, except for caregivers’ reported use of praise. Importantly, the profiles were not characterized by clustering of either positive or negative parenting behaviors. The profiles were rather characterized by intense or lax use of both positive and negative parenting behaviors and by specific combinations of parenting behaviors. This indicates that different and multiple behaviors are important in identifying differences in parenting between families.

Second, the parenting profiles were related to child disruptive behavior and families’ sociodemographic characteristics, which may help us explain why families opt for specific parenting strategies. For example, children with borderline to clinical levels of disruptive behavior were overrepresented in the Harsh Parenting profile (10.1% of the sample). This may indicate a coercive cycle between caregiver and child behavior, in which caregivers’ use of corporal punishment, shouting, and threatening lead to more disruptive child behavior, and vice versa (Pardini, 2008; Patterson, 1976). Moreover, the *average level* of disruptive child behavior was highest in the Low Involvement profile (81.4%). It may be that, in contrast with caregivers in the Harsh Parenting profile, caregivers in this profile did not intensify their parenting strategies in reaction to disruptive child behavior but have “given up.” Indeed, it has been found that in some families, childhood disruptive behavior predicts decreased involvement and poorer supervision over time (Burke et al., 2008). Finally, the overrepresentation of highly educated, majority caregivers in this profile is in line with previous findings on the negative association between socioeconomic status (SES) and harsh parenting (Hoff & Laursen, 2019). Although to date the mechanisms underlying this association are poorly understood, the use of harsh parenting behaviors seems less prevalent among high-SES families.

Disruptive child behavior was lowest in the High Involvement profile (8.4%). In this profile, parenting behavior may be less driven by child behavior, but more by other family characteristics. Low-educated, minority, single, and previously incarcerated caregivers were overrepresented in this profile. These caregivers may compensate for experienced stressors by adjusting their parenting and enhancing the caregiver–child relationship (Arditti et al., 2013; Collins et al., 2000). For example, low-SES families may more frequently experience their neighborhood as unsafe and adjust their monitoring strategies accordingly (Collins et al., 2000). Alternatively, the relation between the parenting profiles and family characteristics may stem from differences in how caregivers from different cultural or SES backgrounds report on their own parenting behavior. How caregivers interpret questionnaire items and what they consider favorable or desirable parenting behavior may differ between caregivers. What is viewed as supportive or positive reinforcement by some may be viewed as permissive or “bribing” by others. This fits with concerns that we know little about the validity of our parenting assessment instruments across families with different backgrounds (e.g., Herbers et al., 2017). Moreover, family characteristics were related to the study families originally participated in. Differences in recruitment strategies likely contributed to the difference in sample composition between studies and possibly, in turn, to the differences between profiles (Lochman & Conduct Problems Prevention Research Group, 1995). The different studies selected families based on different risk factors. In studies that screened families on elevated disruptive child behavior, mainly highly educated, well-off families may respond and/or opt in (Radey & Randolph, 2009). In studies targeting families based on other risk factors, such as socioeconomic disadvantage or caregiver incarceration, families may not necessarily experience disruptive child behavior.

The fact that more than 80% of caregivers in our pooled sample were allocated to the same parenting profile may indicate that the parenting behaviors included in this study alone do not explain differences between families participating in parenting programs. Different child (e.g., ADHD symptoms, executive functioning), caregiver (e.g., mental health, cognitions), and family (e.g., (co)parenting relations, poverty) characteristics may affect how much families benefit from a parenting program, for example because they directly contribute to the etiology or maintenance of disruptive child behavior, interfere with change in parenting or child behavior, and/or are related to caregivers’ engagement in the parenting program (Weeland et al., 2021). Another way forward in explaining heterogeneity in the effectiveness of parenting programs may thus be to explore family profiles based on baseline family characteristics beyond parenting behavior.

Some limitations of our study should be mentioned. First, profiles were solely based on self-reported parenting behaviors. Although self-reported parenting behavior has proven a valid and valuable strategy to assess parenting, it also has limitations (Hawes & Dadds, 2006; Morsbach & Prinz, 2006). Different methods for assessing parenting assess different aspects of the same underlying construct (Hendriks et al., 2018). Complementing self-reported parenting with other measures (e.g., observed parenting) may improve our understanding of different parenting profiles and their correlates. The added benefit of such a multi-method and/or multi-informant method is that it decreases the interdependency between assessment of parenting and child behavior compared with using a single informant. Moreover, as the profiles are based on self-reports, they may also reflect processes underlying how caregivers report on their parenting behaviors, such as feelings of self-efficacy, attributions about child behavior, or (dis)stress (Herbers et al., 2017). For example, caregiver distress has been related to self-critical reports of parenting behavior (Heberle et al., 2015; Herbers et al., 2017). Including such correlates of parenting may further enhance our understanding of differences and similarities between caregivers with different parenting profiles.

Second, and related, parenting behaviors were constructed from different instruments and, although theoretically sound, they were sometimes measured with a single item and reliability for scales with multiple items was sometimes modest. Third, families were allocated to a specific profile based on the probability scores they fitted in that specific profile. Although the profile probabilities for the chosen profile solution were high (between 0.88 and 0.98)—indicating that families could be allocated to a certain profile with high probability—there is still some amount of uncertainty in the classification of individual families. Fourth, all families in our sample participated in a single parenting program (IY) via Dutch research projects and were mostly mothers. Although our sample was diverse and included families of hard-to-reach populations, the extent to which these profiles generalize to other parenting programs (than IY) or other populations (e.g., in research underrepresented populations such as minorities and male caregivers) is unknown. Replication and validation of these parenting profiles in different samples and countries is therefore needed. Finally, in this study, we only have data of the putative mediators and outcomes of the intervention at two time points. We were therefore unable to test time-informed mediation (Kazdin & Nock, 2003) and thus the full baseline target–mediated moderation model (BTMM).

Study strengths include the use of pooled data of different studies, resulting in a larger and more diverse sample than is generally available in single studies and increasing power to assess parenting profiles as a moderator of intervention effectiveness. Moreover, we combined strengths of both variable- and person-centered methods. Although interventions typically target risk factors within individual families (person-centered), most research in this field assesses differences between families (variable-centered) rather than processes within families. The integration of traditional variable-centered and person-centered methods in this study yielded new insights in how families differ in terms of their parenting practices. In addition, it lays the foundation for relevant new questions on how parenting interventions may work similarly or differently for different families.

## Supplementary Information

Below is the link to the electronic supplementary material.

Supplementary file1 (DOCX 124 KB)

Supplementary file2 (DOCX 32 KB)

Supplementary file3 (DOCX 137 KB)

Supplementary file4 (DOCX 25 KB)

Supplementary file5 (DOCX 35 KB)

Supplementary file6 (DOCX 90 KB)
