# Supplementary material for: Exploring Parenting Profiles to Understand Who Benefits from the Incredible Years Parenting Program
Source: Prev Sci. 2022 Mar 19;24(2):259–70. doi: 10.1007/s11121-022-01364-6 (PMC9938070; doi:10.1007/s11121-022-01364-6)
Supplement: Supplementary file 1 — Supplementary file1 (DOCX 124 KB) [file 11121_2022_1364_MOESM1_ESM.docx]

**Online Resource 1**

**Procedures and recruitment**

In Study #1 *(n* = 144), families with a 4-year-old child from different towns and cities in a Dutch province were sent a screening list via the *Office for Screening and Vaccination*. Families who scored ≥ 80th percentile of the Aggressive Behavior scale of the Child Behavior Checklist 1½–5 (CBCL; Achenbach, Rescorla, & Maruish, 2004) were invited to participate (Posthumus et al., 2012). In Study #2 (*n* = 99), participants were recruited through nationwide screening for incarcerated and recently released mothers or via organizations working with (formerly) incarcerated women (Menting et al., 2014). Due to a smaller number of mothers ending their prison sentences, and thus lower number of potential participants, randomization was temporarily suspended in this study. During two of the six recruitment periods all participants were assigned to the intervention condition to ensure the minimum number of caregivers per IY group. Study #2 included multiple children per family. We selected per family the child whose age was closest to the mean target age group of IY (7.5 years). In Study #3 (*n* = 156), participants were either referred for disruptive child behavior to outpatient clinics for child and adolescent psychiatry or actively recruited in socioeconomically deprived neighborhoods (Leijten et al., 2017). In Study #4 (*n* = 387), families with children ages 4–8 years of four municipalities were screened and recruited through community records via two Dutch regional health care organizations. Families scoring ≥ 75th percentile on the intensity scale of the Eyberg Child Behavior Inventory (Eyberg et al., 1999), were invited to participate.

Table 1.1.

*Description of the Included Studies*

| **Study** | **Authors (year)** | **Design** | **Control condition** | **Recruitment strategy** | **Parenting measure** | ***n***^a^ | **Retention**  **%** | **Child age**  **(*M*)** | **%boys** | **Disruptive behavior**  **problems**  **pre-intervention**  **%**  **> 75^th^ percentile** | **% low educated** | **% single parent** | **% Ethnic minority** |
| --- | --- | --- | --- | --- | --- | --- | --- | --- | --- | --- | --- | --- | --- |
| 1 | Posthumus et al. (2012) | Case-control | No active intervention | Screening on disruptive child behavior via Office for Screening and Vaccination | PPI | 141 | 98.6 | 4.43 | 70.9 | 71.9 | 11.0 | 10.8 | 4.3 |
| 2 | Menting et al., (2014) | Part RCT and part no randomization | No active intervention | Recruitment via penitentiary institutions and via organizations for formerly incarcerated women | APQ | 92 | 71.3 | 6.49 | 45.5 | 34.4 | 73.9 | 45.5 | 78.5 |
| 3 | Leijten et al. (2017) | RCT | Wait-list | Screening on disruptive child behavior via outpatient psychiatric clinics and schools, open recruitment | PPI | 129 | 73.7 | 5.60 | 61.9 | 61.2 | 76.9 | 7.1 | 64.7 |
| 4 | Weeland & Chhangur et al. (2017) | RCT | No active intervention | Screening on disruptive child behavior via Office for Screening and Vaccination | PPI | 385 | 94.1 | 6.31 | 55.3 | 88.3 | 21.2 | 10.1 | 14.0 |
|  |  |  |  |  |  |  |  |  |  |  |  |  |  |

*Note*. a. Included in analyses in current study. With complete data on parenting behavior pre-intervention.

**Flow Charts per Study**

**Flow Diagram Study 1**

Signed consent (n=144)

Assessed for eligibility (n= 8632)

## Post-test

## Enrollment

Excluded (n = 8488):

♦Not meeting inclusion criteria (n=8167)

♦Declined to participate (n = 321)

**Flow Diagram Study 2**

Analysed in profile analysis pre-intervention (n=70)
♦Excluded from analysis (n=2) due to missings on data needed for profile analysis

Analysed in profile analysis post-intervention (n=69)

♦ Excluded from analysis (n=2) due to missings on data needed for profile analysis

Discontinued participation in study (n=1)

Discontinued participation in study (n=1)

Discontinued intervention (n=2) (these families were invited to continue participation in the study)

Analysed in profile analysis pre-intervention (n=71)
♦Excluded from analysis (n=1) due to missings on data needed for profile analysis

Analysed in profile analysis post-intervention (n = 70)

♦Excluded from analysis (n=1) due to missings on data needed for profile analysis

## Analysis

## Allocation

Allocated to intervention (n= 72)

Allocated to control group (n= 72)

## Analysis

Analysed in profile analysis pre-intervention (n=24)

♦ Excluded from analysis (n=3) due to missings on data needed for profile analysis

Analysed in profile analysis post-intervention
(n=18)

Discontinued participation in study (n=9)

Analysed in profile analysis pre-intervention (n=68)

♦ Excluded from analysis (n=18) due to missings on data needed for profile analysis

Analysed in profile analysis post-intervention
(n=54)

♦ Excluded from analysis (n=4) due to missings on data needed for profile analysis

Discontinued participation in study (n=28)

Discontinued intervention (n=15) (these families were invited to continue participation in the study)

Allocated to intervention (n=86)

♦Received allocated intervention (n=55)

♦ Did not receive allocated intervention (n=31)

Allocated to control group (n=27)

## Post-test

## Allocation

Excluded (n=70)

♦Did not meet inclusion criteria (n=17)

♦Declined to participate (n=23)

♦Unable to contact (n=30)

Signed consent (n=113)

Assessed for eligibility (n=183)

## Enrollment

**Flow Diagram Study 3**

## Post-test

## Enrollment

## Allocation

Randomized (n= 154)

Excluded (n= 167)

♦ Not meeting inclusion criteria (n= 3)

♦  Declined to participate (n= 164)

Assessed for eligibility (n= 321)

Allocated to control group (n= 47)

Allocated to intervention (n= 107)

♦Received allocated intervention (n= 104)

♦ Did not receive allocated intervention (n= 3)

## Analysis

Analysed in profile analysis pre-intervention (n= 102)
♦Excluded from analysis (n= 5) due to missings on data needed for profile analysis

Analysed in profile analysis post-intervention (n= 71)

Lost to follow-up (n= 36)

Discontinued intervention (n= 20) (these families were invited to continue participation in the study)

Analysed in profile analysis pre-intervention (n= 27)
♦Excluded from analysis (n= 1) due to missings on data needed for profile analysis

Analysed in profile analysis post-intervention (n= 44)

Lost to follow-up (n= 3)

**Flow Diagram Study 4**

Excluded (n= 6263):

♦Not meeting inclusion criteria (n = 4405)
♦Declined to participate (n= 463)
♦Unable to contact (n = 543)

Signed consent (n= 387)

Assessed for eligibility (n= 5876)

## Post-test

## Enrollment

## Allocation

Discontinued participation in study (n= 8

Analysed in profile analysis pre-intervention (n= 189)

♦Excluded from analysis (n= 1) due to missings on data needed for profile analysis

Analysed in profile analysis post- intervention (n= 182)

## Analysis

Analysed in profile analysis pre-intervention (n= 196)

♦Excluded from analysis (n= 1) due to missings on data needed for profile analysis

Analysed in profile analysis post- intervention (n= 182)

Discontinued participation in study (n= 15)

Discontinued intervention (n= 18) (these families were invited to continue participation in the study)

Allocated to intervention (n= 190)

Allocated to intervention (n= 197)

♦Received allocated intervention (n= 146)

♦ Did not receive allocated intervention

(n= 44)
