# Supplementary material for: Exploring Parenting Profiles to Understand Who Benefits from the Incredible Years Parenting Program
Source: Prev Sci. 2022 Mar 19;24(2):259–70. doi: 10.1007/s11121-022-01364-6 (PMC9938070; doi:10.1007/s11121-022-01364-6)
Supplement: Supplementary file 2 — Supplementary file2 (DOCX 32 KB) [file 11121_2022_1364_MOESM2_ESM.docx]

**Online Resource 2.**

**Harmonizing procedure parenting scales**

Parenting behavior was assessed using the Parenting Practices Interview (Webster Stratton, 2001) (used in study #1, #3 and #4) and the Alabama Parenting Questionnaire (Essau et al., 2006) (used in study #2). Both instruments are well-validated to assess parenting behavior and effectiveness of parenting programs (Dadds et al., 2003; Leijten et al., 2018). Following the procedure by Leijten and Gardner et al. (2018), seven parenting behaviors were selected and defined: corporal punishment; threatening; laxness, shouting, praise; tangible rewards; and monitoring. For both instruments we selected items best representing these aspects of parenting, balancing reliability of the scales (using McDonald’s omega (ω), Hayes & Coutts, 2020), keeping as much items as possible and maximizing comparability between instruments. See for correlations between parenting behaviors and disruptive child behavior Table 1.1.

Corporal punishment was defined as any physical punishment such as slapping or spanking the child. The APQ, for example, asks: *‘You slap your child when he/she has done something wrong’*) The PPI asks: ‘*How often do you do each of the following things when your child misbehaves? Slap or hit your child*’.

Threatening was defined as threatening the child with punishment but not directly punishing him/her. The APQ asks ‘*You threaten to punish your child and then do not actually punish him/her*’. The PPI, for example, asks ‘*How often do you do each of the following things when your child misbehaves? Threaten to punish him/her (but not really punish him/her)’*,

Laxness was defined as intending to discipline the child, but not actually following it through. The APQ asks: ‘*Your child talks you out of being punished after he/she has done something wrong*’ and the PPI asks: ‘*How often does your child get away with things that you feels/he should have been disciplined for?’*.

Shouting was defined as raising of the voice, shouting, scolding, and the use of mean or foul language toward the child. The APQ asks ‘*You yell or scream at your child when he/she has done something wrong*’. The PPI asks ‘*How often do you do each of the following things when your child misbehaves? Raise your voice (scold or yell)*’.

Praise was defined as verbal rewards, giving compliments in response to the child's behavior. The APQ asks: ‘*You compliment your child when he/she does something well*’. The PPI asks: ‘*In general, how often do you praise or compliment your child when your child behaves well or does a good job?*’.

Tangible rewards was defined as nonverbal rewards, such as privileges or stickers on a chart. The APQ asks ‘*You reward or give something extra to your child for obeying you or behaving well*’ and the PPI, for example asks; ‘*You reward or give something extra to your child for obeying you or behaving well’.*

Monitoring was defined as parental supervision and knowledge of the child's whereabouts when the child was out of the parents’ sight, including knowing who the child's friends are. For example, the APQ asks ‘*Your child is out with friends you don’t know*’(this item was reverse coded) and the PPI asks ‘*What percentage of your child’s friends do you know well*?’.

Table 2.1.

*Correlations between Parenting Behaviors and Child Behavior*

|  |  | 1 | 2 | 3 | 4 | 5 | 6 | 7 | 8 |
| --- | --- | --- | --- | --- | --- | --- | --- | --- | --- |
| 1 | Threatening T1 | - | .19** | .05 | .33** | .18** | -.10** | .25** | .09** |
| 2 | Corporal punishment T1 | - | - | -.07 | .16** | .16** | -.08* | .31** | .05 |
| 3 | Monitoring T1 | - | - | - | -.02 | .22** | .10** | -.10** | -.12** |
| 4 | Laxness T1 | - | - | - | - | .16** | -.08* | .24** | .17** |
| 5 | Tangible rewards T1 | - | - | - | - | - | .32** | -.05 | -.14** |
| 6 | Praise T1 | - | - | - | - | - | - | -.13** | -.09* |
| 7 | Shouting T1 | - | - | - | - | - | - | - | .34** |
| 8 | Disruptive child behavior T1 | - | - | - | - | - | - | - | - |

*Note.* * *p* < .05; ** *p* <.01

**Items used per scale:**

**Corporal punishment**

**APQ** (used in 1 study: 3 items: 31, 33, 36, α =.63, ω= .82)

31. You spank your child with your hand when he/she has done something wrong

33. You slap your child when he/she has done something wrong

36. You hit your child with a belt, switch, or other object when he/she has done something wrong

**PPI** (used in 3 studies: 6 items: 1h, 1i, 2h, 2i, 3h, 3i, α = .83, ω= .84)

1. How often do you do each of the following things when your child misbehaves?

1.h Give your child a spanking

1.i Slap or hit your child (but not spanking)

2. If your child hit another child, how likely is it that you would discipline your child in the following ways?

2.h Give your child a spanking

2.i Slap or hit your child (but not spanking)

3. If your child refused to do what you wanted him/her to do, how likely is it that you would use each of the following discipline techniques?

3.h Give your child a spanking (in #10: give your child a smack)

3.i Slap or hit your child (but not spanking) (in #10: slap or hit your child)

**Threatening**

**APQ** (used in 1 study: 1 item: 3)

3. You threaten to punish your child and then do not actually punish him/her.

**PPI** (used in 3 studies: 3 items: 1d, 2d, 3d, α = .77 , ω= .78)

1. How often do you do each of the following things when your child misbehaves?

1.d Threaten to punish him/her (but not really punish him/her)

2. If your child hit another child, how likely is it that you would discipline your child in the following ways?

2.d Threaten to punish him/her (but not really punish him/her)

3. If your child refused to do what you wanted him/her to do, how likely is it that you would use each of the following discipline techniques?

3.d. Threaten to punish him/her (but not really punish him/her)

**Laxness**

**APQ** (used in 1 study: 1 item: 7)

7. Your child talks you out of being punished after he/she has done something wrong

**PPI** (used in 3 studies: 1 item: 5c)

5.c How often does your child get away with things that you feels/he should have been disciplined for?

**Shouting**

**APQ** (used in 1 study: 1 item: 37)

37. You yell or scream at your child when he/she has done something wrong.

**PPI** (used in 3 studies: 5 items: 1b, 2b, 3b, 5e, 5f, α = .71, ω= .74)

1. How often do you do each of the following things when your child misbehaves?

1.b Raise your voice (scold or yell)

2. If your child hit another child, how likely is it that you would discipline your child in the following ways?

2.b Raise your voice (scold or yell)

3. If your child refused to do what you wanted him/her to do, how likely is it that you would use each of the following discipline techniques?

3.b Raise your voice (scold or yell)

5.e How often do you show anger when you discipline your child?

5.f How often do arguments with your child build up and you do or say things you don’t mean to?

**Praise**

**APQ** (3 items: 13, 16, 27, α = .71, ω=.76)

13. You compliment your child when he/she does something well

16. You praise your child if he/she behaves well

27. You let your child know that you appreciate the child’s help with chores

**PPI** (used in 3 studies: 3 items: 6b, 7, 8a, α = .67, ω=.74)

6. In general, how often do you this when your child has done a good job? (Not available in study #3)

6.b. Praise or compliment your child

7. In a regular week, how often do you reward or praise your child when your child behaves well or does a good job at home or at school?

8.a Within the last 2 days how many times did you praise or compliment your child for anything she/he did well?

**Tangible rewards**

**APQ** (1 item: 5)

5. You reward or give something extra to your child for obeying you or behaving well

**PPI** (4 items: 6d, 6e, 6f, & 8b, α = .57, ω= .63 used in 3 datasets)

6. In general, how often do you this when your child has done a good job? (Not available in study #3)

6.d. Buy something for him/her (such as special food, a small toy) or give him/her money.

6.e Give points or stars on a chart

6.f Give him/her an extra privilege (such as cake, go to the movies, special activity)

8.b Within the last 2 days how many times did you give him or her something extra, like a small gift, privileges, or a special activity with you, for something he/she did well.

**Monitoring**

**APQ** (6 items: 10r, 17r, 19r, 21r, 30r & 32r, α = .64, ω= .67)

10. Your child stays out in the evening past the time he/she is supposed to be home (*reverse coded*)

17. Your child is out with friends you don’t know (*reverse coded*)

19. Your child goes out without a set time to be home (*reverse coded*)

21. Your child is out after dark without an adult with him/her (*reverse coded*)

30. Your child comes home from school more than an hour past the time you expect him/her (*reverse coded*)

32. Your child is at home without adult supervision (*reverse coded*)

**PPI** (used in 3 studies: 5 items: 12r, 13r, 14a, 14b, 14c, α = .55, ω= .64)

12. About how many hours in the last 24 hours did your child spend at home without adult supervision? (*Reverse coded*)

13. Within the last 2 days, about how many total hours was your child involved in activities outside your home without adult supervision? (*Reverse coded*)

14.a What percentage of the time do you know where your child is when s/he is away from your direct supervision?

14.b What percentage of the time do you know exactly what your child is doing when s/he is away from you?

14.c What percentage of your child’s friends do you know well?

**References**

Dadds, M. R., Maujean, A., & Fraser, J. A. (2003). Parenting and conduct problems in children: Australian data and psychometric properties of the alabama parenting questionnaire. *Australian Psychologist*, *38*(3), 238–241. https://doi.org/10.1080/00050060310001707267

Essau, C. A., Sasagawa, S., & Frick, P. J. (2006). Psychometric Properties of the Alabama Parenting Questionnaire. *Journal of Child and Family Studies*, *15*(5), 595–614. https://doi.org/10.1007/s10826-006-9036-y

Hayes, A. F., & Coutts, J. J. (2020). Use Omega Rather than Cronbach’s Alpha for Estimating Reliability. But…. *Communication Methods and Measures*, *14*(1), 1–24. https://doi.org/10.1080/19312458.2020.1718629

Leijten, P., Gardner, F., Landau, S., Harris, V., Mann, J., Hutchings, J., Beecham, J., Bonin, E.-M., & Scott, S. (2018). Research Review: Harnessing the power of individual participant data in a meta-analysis of the benefits and harms of the Incredible Years parenting program. *Journal of Child Psychology and Psychiatry*, *59*(2), 99–109. https://doi.org/10.1111/jcpp.12781

Webster-Stratton, C. (2001). Parenting practices interview. *Unpublished Assessment Instrument*.
