# Supplementary material for: Exploring Parenting Profiles to Understand Who Benefits from the Incredible Years Parenting Program
Source: Prev Sci. 2022 Mar 19;24(2):259–70. doi: 10.1007/s11121-022-01364-6 (PMC9938070; doi:10.1007/s11121-022-01364-6)
Supplement: Supplementary file 3 — Supplementary file3 (DOCX 137 KB) [file 11121_2022_1364_MOESM3_ESM.docx]

**Online Resource 3.**

**Distribution of Parenting and Child Behavior Measures at Baseline**


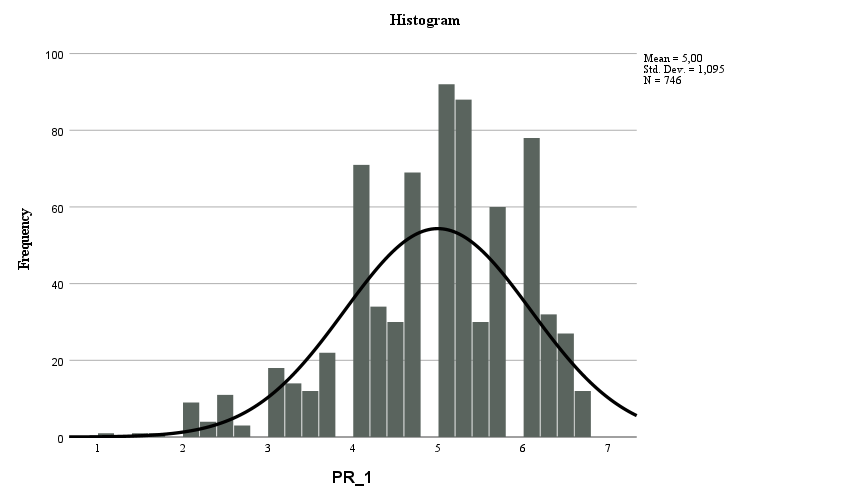


*Figure 3.1.* Distribution of Baseline Scores on Praise


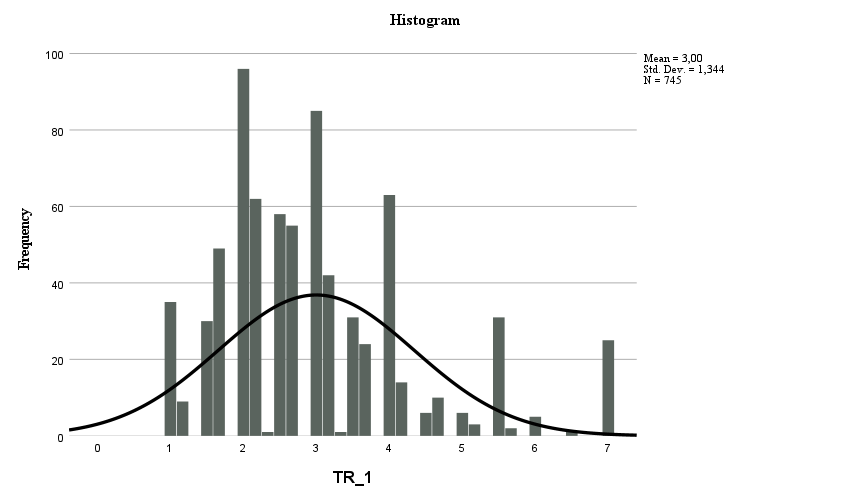


*Figure 3.2*. Distribution Baseline Scores on Tangible Rewards


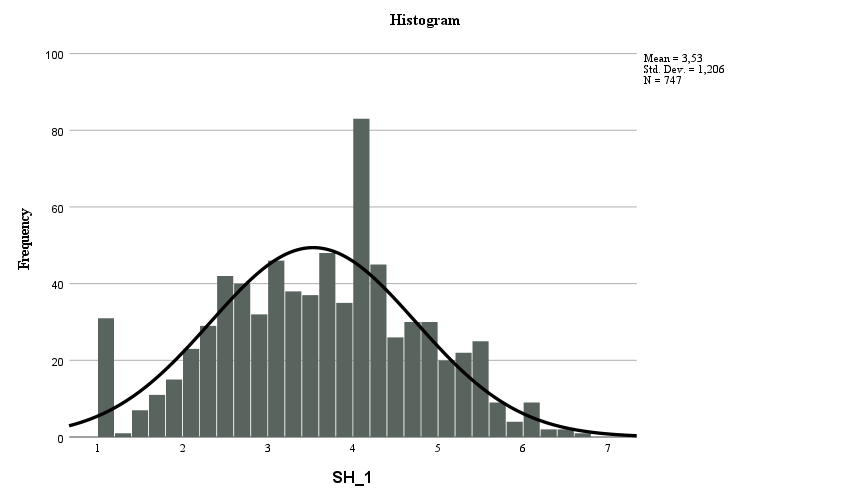


*Figure 3.3.* Distribution of Baseline Scores on Shouting


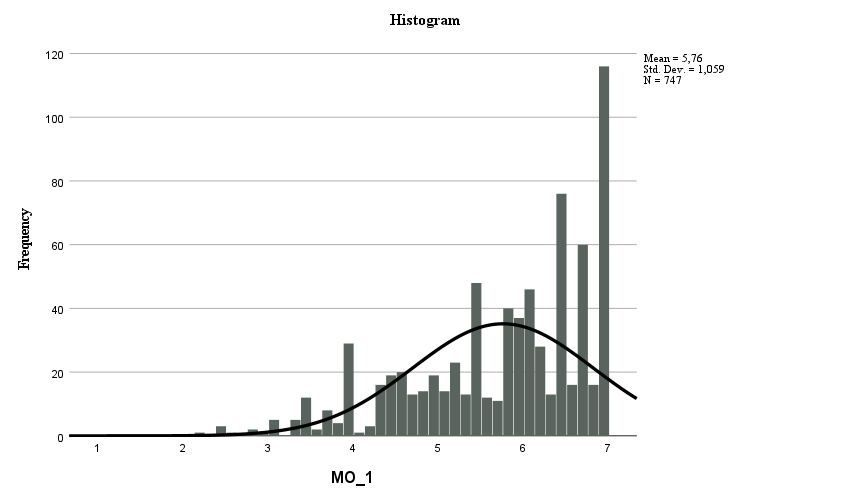


*Figure 3.4.* Distribution of Baseline Scores on Monitoring


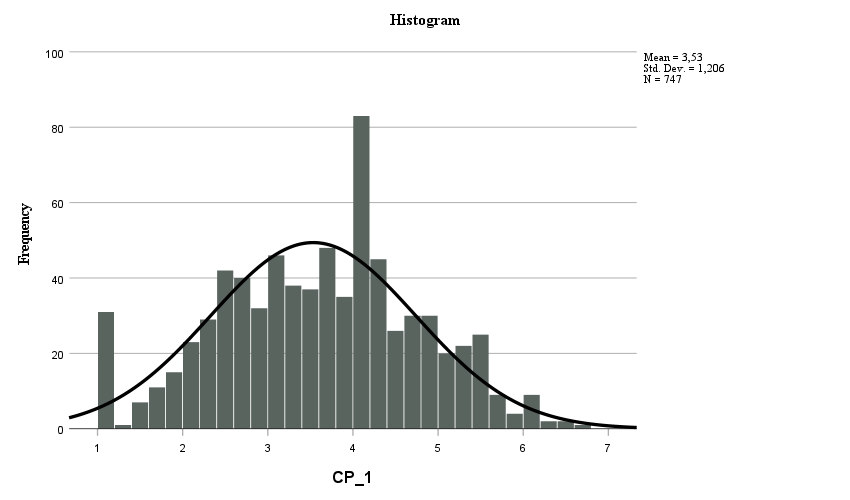


*Figure 3.5.* Distribution of Baseline Scores on Corporal Punishment


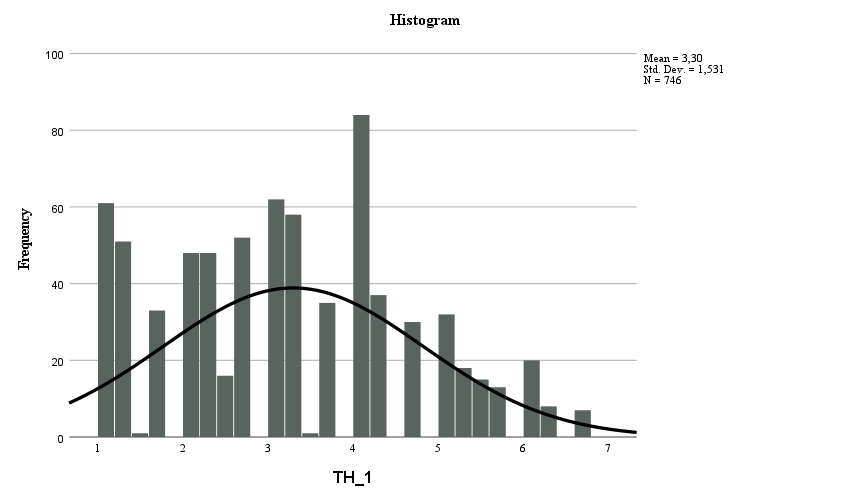


*Figure 3.6.* Distribution of Baseline Scores on Threatening


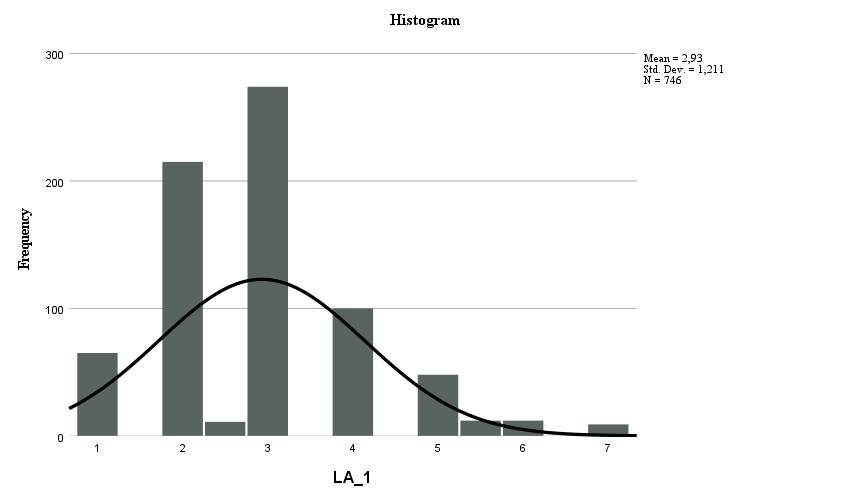


*Figure 3.7.* Distribution of Baseline Scores on Laxness


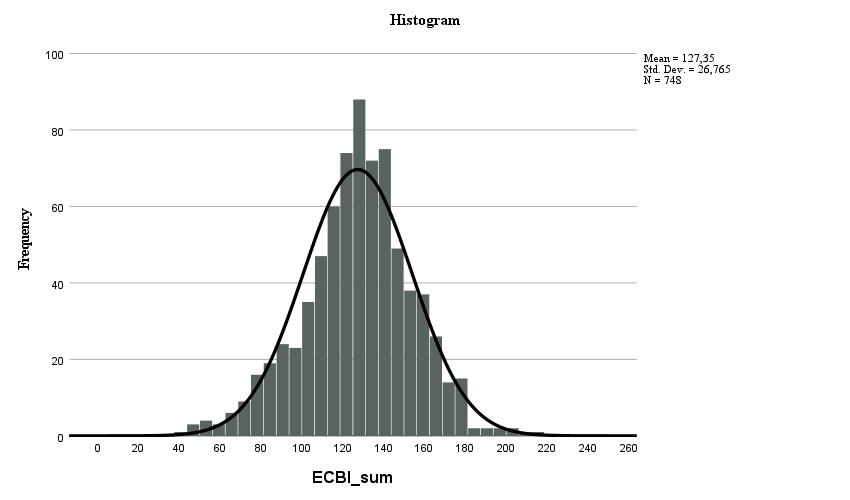


*Figure 3.8.* Distribution of Baseline Sumscores on Disruptive Child Behavior
