# Supplementary material for: Exploring Parenting Profiles to Understand Who Benefits from the Incredible Years Parenting Program
Source: Prev Sci. 2022 Mar 19;24(2):259–70. doi: 10.1007/s11121-022-01364-6 (PMC9938070; doi:10.1007/s11121-022-01364-6)
Supplement: Supplementary file 4 — Supplementary file4 (DOCX 25 KB) [file 11121_2022_1364_MOESM4_ESM.docx]

| **# profiles** | **AIC** | **BIC** | **Lo-Mendell-Rubin adjusted LRT test** | | **Entropy** | **Lowest class probability** |
| --- | --- | --- | --- | --- | --- | --- |
| 1 | 14560.464 | 14623.978 | - |  | - | - |
| 2 | 14097.736 | 14197.543 | 469.745 | *p* =.005 | .981 | .950 |
| 3 | 13847.235 | 13983.336 | 261.500 | *p* =.003 | .839 | .820 |
| 4 | 13738.507 | 13910.902 | 122.387 | *p* =.084 | .835 | .804 |
| 5 | 13611.540 | 13820.228 | 100.130 | *p* =.741 | .868 | .780 |
| 6 | 13620.710 | 13865.691 | 28.375 | *p* =.594 | .784 | .746 |
|  |  |  |  |  |  |  |

**Online Resource 4.**

**Parenting Profiles Post-Intervention**

Table 4.1.

*Result of Profile Analyses*

Table 4.2.

*Descriptives for and Differences Between the Two Post Intervention Parenting Profiles*

|  | **Profile 1**  **(*n* = 645)** | | | **Profile 2**  **(*n* = 45)** | | |  |
| --- | --- | --- | --- | --- | --- | --- | --- |
|  | ***M*** | ***SE*** | **95%**  **CI** | ***M*** | ***SE*** | **95%**  **CI** |  |
| Corporal punishment T2* | 1.25 | 0.02 | 1.23 – 1.28 | 3.60 | 0.14 | 3.35 – 3.89 |  |
| Shouting T2* | 3.10 | 0.04 | 3.02 – 3.19 | 4.09 | 0.18 | 3.73 – 4.43 |  |
| Praise T2 | 5.17 | 0.04 | 5.09 – 5.26 | 5.03 | 0.17 | 4.69 – 5.35 |  |
| Tangible rewards T2* | 3.12 | 0.05 | 3.04 – 3.23 | 3.89 | 0.24 | 3.42 – 4.34 |  |
| Monitoring T2 | 5.67 | 0.04 | 5.57 – 5.75 | 6.07 | 0.15 | 5.75 – 6.35 |  |
| Threatening T2* | 2.58 | 0.05 | 2.48 – 2.70 | 3.72 | 0.22 | 3.30 – 4.14 |  |
| Laxness T2* | 2.72 | 0.40 | 2.63 – 2.80 | 3.25 | 0.24 | 2.80 – 3.74 |  |
| Disruptive child behavior T2  (ECBI sum score)* | 119.35 | 1.03 | 117.98 – 122.03 | 118.48 | 4.16 | 110.21 – 126.55 |  |
|  | **Profile 1** | | | **Profile 2** | | |  |
|  |  | **%** |  |  | **%** |  |  |
| Child disruptive behavior > 90^th^ percentile T2^a^ |  | 33.5 |  |  | 40.9 |  |  |
| Single caregiver* |  | 12.2 |  |  | 29.5 |  |  |
| Low educated* |  | 28.5 |  |  | 55.0 |  |  |
| Ethnic status* |  | 23.6 |  |  | 58.1 |  |  |
|  |  |  |  |  |  |  |  |

*Note.* ^a^ Based on age- and sex-specific Dutch norm scores (Weeland, Van Aar, & Overbeek, 2018);

*significant difference between profiles: Corporal punishment: *B* = 0.11 *SE* = 0.03, *p* < .01; Shouting: *B* = 0.25, *SE* = .05, *p* < .001; Praise: *B* = -0.08, *SE* = 0.04, *p* = .82; Tangible rewards: *B* = 0.19, *SE* = 0.06, *p* < .01; Monitoring: *B* = 0.03, *SE* = 0.04, *p* = .41; Threatening: *B* = 0.64, *SE* =0 .06, *p* < .001; Laxness: *B* = 0.23, *SE* = 0.05, *p* < .001; Education: *B* = -0.29, *SE* = 0.09, *p*<.01; Minority status: *B* = 0.37, *SE* = 0.08, *p*<.001, Single caregiver: *B* = 0.17, *SE* = 0.07, *p*=.02; Disruptive behavior: *B* = -0.50, *SE* = 4.19, *p* =.91; ECBI normscore: *B* = 0.11, *SE* = 0.22, *p* = .61

**Changes in Parenting Profiles.**

The sequential profiles analyses with post-intervention data showed that AIC and BIC decreased when the number of profiles increased, indicating increased model fit with more profiles. The LRT test was significant up to five profiles meaning that the increase in model fit was no longer significant when we went from five to six profiles. Entropy was above .950 for the two-profile solution (indicating good classification quality), but under .900 for the other solution. Class probabilities were above .940 in the two-profile solution but fell under .850 for other profile solutions (see Table 4.1). Moreover, they included small profiles (< 6% of the sample). A 2-profile solution was therefore chosen (entropy = .975, class probabilities > .940).

The two parenting profiles significantly differed on all parenting behaviors, except monitoring and praise, and on all sociodemographic variables, except for disruptive child behavior (*Chi*^2^ = 0.00, *df* = 0, *p* <.001; RMSEA <.001; CFI = .1.00; SRMR =<.001 (saturated models), see results in the note of Table 4.2 and 95% confidence intervals Table 4.2). Post-intervention profile 1 (*n* = 644, average latent class probability = .998) closely resembles the pre-intervention profile *‘Low Involvement’*. Indeed, most caregivers in this profile were also allocated to this profile pre-intervention (85.62%). Post-intervention profile 2 (*n* = 46, average latent class probability = .950) closely resembles the pre-intervention profile ‘*Harsh Parenting’*. Indeed, about half of the caregivers in this profile were also allocated to this profile pre-intervention (51.3%). The clustering of parenting behaviors thus seemed relatively consistent over time.

Assuming that post-intervention profiles 1 and 2 are indeed comparable to respectively the pre-intervention *Low Involvement* and *Harsh Parenting* profiles, we see the following changes in profile membership: Of caregivers allocated to the pre-intervention *Low Involvement* profile 1 2.7% changed profile membership from pre- to post-intervention. This profile thus seemed relatively stable over time. Of caregivers allocated to the pre-intervention *Harsh Parenting* profile 73.5% changed profile membership (of which 88.1% were allocated to the *Low Involvement* post-intervention). The pre-intervention *High Involvement* profile was not found post-intervention. Of caregivers allocated to this profile pre-intervention 73.5% were allocated to post-intervention profile *Low Involvement*. These latter two profiles thus seemed less stable over time. The relative low retention rate in the *Harsh Parenting* profile (i.e., for 28.6% of caregivers in this profile there was no post-intervention data available vs. 9.5 and 10.5% for caregivers in the other profiles) may have contributed to the disappearance of this profile.

We assessed if changes in parenting profile membership from pre- to post-intervention was predicted by pre-intervention profile and condition (IY vs. control) (controlling for the study from which participants originated since these were not equally distributed across profiles). In the control condition 12% of caregivers changed profile membership whereas in the intervention condition this was 21%. Pre-intervention parenting profile predicted change to a different profile, but condition did not (*Chi*^2^ = 0.00, *df* = 0, *p* <.001; RMSEA <.001; CFI = .1.00; SRMR =<.001 [saturated models], profile: *B* = 0.42, *SE* = .03, *p*<.001; condition: *B*=0.04, *SE* = 0.02, *p* = .06). We also checked for a possible interaction between profile and condition by adding an interaction between condition and pre-intervention profile. However, this interaction was not significant (condition-×-profile: *B*= -0.02, *SE*=0.02, *p*=.46).
