# Supplementary material for: Exploring Parenting Profiles to Understand Who Benefits from the Incredible Years Parenting Program
Source: Prev Sci. 2022 Mar 19;24(2):259–70. doi: 10.1007/s11121-022-01364-6 (PMC9938070; doi:10.1007/s11121-022-01364-6)
Supplement: Supplementary file 5 — Supplementary file5 (DOCX 35 KB) [file 11121_2022_1364_MOESM5_ESM.docx]

**Online Resource 5.**

**Baseline Target Moderation Model**

Parenting profile at baseline

Disruptive child behavior post-intervention

Condition

(control vs. intervention)

*Figure 5.1.*

Graphic Illustration of Theoretical Model: Baseline Target Moderation Model

**
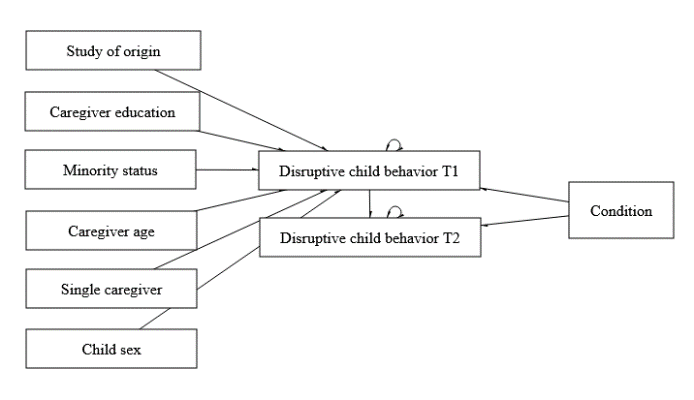
**

*Figure 5.2.*

Illustration of Path Model Used to Test Effects of Experimental Condition on Disruptive Child Behavior at T2, Controlling for Co-variates and Disruptive Child Behavior at T1.

*Note*. Parenting profile is used as a grouping variable to assess whether the path from condition to disruptive child behavior at T2 (post-intervention) was different per profile.
