# Supplementary material for: Exploring Parenting Profiles to Understand Who Benefits from the Incredible Years Parenting Program
Source: Prev Sci. 2022 Mar 19;24(2):259–70. doi: 10.1007/s11121-022-01364-6 (PMC9938070; doi:10.1007/s11121-022-01364-6)
Supplement: Supplementary file 6 — Supplementary file6 (DOCX 90 KB) [file 11121_2022_1364_MOESM6_ESM.docx]

**Online Resource 6.**

**Descriptive Statistics Parenting and Child Behavior and Differential Effectiveness**

A path model in which condition (IY vs. intervention) predicted disruptive behavior post-intervention, controlling for disruptive child behavior pre-intervention, study from which families originated, child age, caregiver sex, education, minority status, and being a single caregiver), was estimated. The model had excellent fit (*Chi*^2^ = 3.952, *df* = 5, *p* = .556; RMSEA < .001; CFI = 1.00; SRMR = .013). These results indicate that condition did not have a significant effect on disruptive child behavior at pre-intervention but did have a significant effect on disruptive child behavior post-intervention. Caregivers in the intervention group reported less child disruptive behavior (*B* = -0.228, *SE* = 0.037, *p* <.001; Cohen’s *d* = −0.27) at post-test compared to caregivers in the control group (see for means Table 6.1).

*Chi^2^* decreased when the path from condition to disruptive behavior post-intervention was estimated freely (*Chi^2^* = 3.188), compared to when this path was constrained to be equal across profiles (*Chi^2^* = 3.952). This indicates better model fit in the freely estimated model. However, this difference was not significant (difference = -0.806, *p* = 1.000). We therefore found no evidence that the effect of IY on disruptive child behavior was different across profiles (see results of the path models, Table 6.2).

Table 6.1.

*Means and Standard Deviations of Parenting and Child Outcomes at Pre-Intervention (T1) and Post Intervention (T2)*

|  | **Intervention** | |  | **Control** | |  |  |
| --- | --- | --- | --- | --- | --- | --- | --- |
|  | ***M*** | ***SD*** | **Range** | ***M*** | ***SD*** | **Range** |  |
| Disruptive child behavior T1 | 3.51 | .77 | 1.00 – 5.73 | 3.57 | 1.52 | 1.31 – 5.92 |  |
| Disruptive child behavior T2 | 3.22 | .70 | 1.03 – 5.11 | 3.47 | .71 | 1.33 – 5.92 |  |
|  |  |  |  |  |  |  |  |
| Threatening T1 | 3.35 | 1.53 | 1.00 – 7.00 | 3.23 | 1.52 | 1.00 – 7.00 |  |
| Threatening T2 | 2.44 | 1.29 | 1.00 - 7.00 | 2.92 | 1.47 | 1.00 – 7.00 |  |
|  |  |  |  |  |  |  |  |
| Corporal punishment T1 | 1.59 | .78 | 1.00 – 5.33 | 1.48 | .71 | 1.00 – 4.67 |  |
| Corporal punishment T2 | 1.38 | .70 | 1.00 – 6.50 | 1.44 | .76 | 1.00 – 5.50 |  |
|  |  |  |  |  |  |  |  |
| Monitoring T1 | 5.81 | 1.07 | 2.20 – 7.00 | 5.70 | 1.04 | 2.50 - 7.00 |  |
| Monitoring T2 | 5.77 | 1.03 | 1.60 – 7.00 | 5.62 | 1.00 | 2.80 – 7.00 |  |
|  |  |  |  |  |  |  |  |
| Laxness T1 | 2.98 | 1.28 | 1.00 – 7.00 | 2.85 | 1.11 | 1.00 – 7.00 |  |
| Laxness T2 | 2.69 | 1.04 | 1.00 – 7.00 | 2.83 | 1.08 | 1.00 – 7.00 |  |
|  |  |  |  |  |  |  |  |
| Tangible rewards T1 | 3.07 | 1.40 | 1.00 – 7.00 | 2.91 | 1.27 | 1.00 – 7.00 |  |
| Tangible rewards T2 | 3.49 | 1.38 | 1.00 – 7.00 | 2.83 | 1.17 | 1.00 – 7.00 |  |
|  |  |  |  |  |  |  |  |
| Praise T1 | 4.97 | 1.15 | 1.00 -7.00 | 5.03 | 1.01 | 2.00 – 7.00 |  |
| Praise T2 | 5.35 | 1.12 | 1.50 -7.00 | 4.94 | 1.04 | 1.00 – 7.00 |  |
|  |  |  |  |  |  |  |  |
| Shouting T1 | 3.53 | 1.23 | 1.00 -7.00 | 3.55 | 1.18 | 1.00 – 7.00 |  |
| Shouting T2 | 3.04 | 1.13 | 1.00 -6.40 | 3.32 | 1.15 | 1.00 – 6.80 |  |

*Note.* All mean scores on a 7-point scale

|  | ***B*** | ***SE*** | ***p*** | **Lower**  **boundary 95% CI** | **Upper boundary**  **95% CI** | **Cohen’s *d*** |
| --- | --- | --- | --- | --- | --- | --- |
| Complete sample | -0.228 | 0.037 | <.001 | -0.305 | -0.145 | -.27 |
| Low Involvement profile | -0.221 | 0.039 | <.001 | -0.298 | -0.143 | -.26 |
| High Involvement profile | -0.176 | 0.180 | .331 | -0.532 | 0.215 | -.39 |
| Harsh Parenting profile | -0.265 | 0.143 | .065 | -0.559 | 0.015 | -.44 |

Table 6.2.

*Effect of Condition (IY vs. Control) on Disruptive Child Behavior Post-Intervention for Complete Sample and Per Pre-Intervention Parenting Profile*

*Additional analyses*

As an additional test of the validity of using parenting profiles over individual parenting behaviors as moderators of the intervention effect, we also explored whether individual parenting behaviors pre-intervention moderated intervention effects on disruptive child behavior post-intervention. The model had excellent model fit (*Chi*^2^ = 10.630, *df* = 8, *p* = .224; RMSEA =.022; CFI = 1.000; SRMR = .008). In this model the direct effect of condition was no longer significant. Moreover, none of the parenting behaviors (except for laxness) had a significant effect on child behavior post-intervention, either direct or in interaction with condition. The interaction effect of condition and laxness on disruptive behavior post-intervention was significant: The effect of condition on disruptive child behavior post-intervention was larger when laxness at pre-intervention was low. However, the CIs of this effect contained 0 across the range of laxness in the sample. We therefore conclude we found no evidence of a differential effect of IY on disruptive behavior based on individual parenting behaviors pre-intervention**.**

Table 6.3.

*Effect of Condition (IY vs. Control), Individual Parenting Behaviors and Condition-x-Parenting Behaviors on Disruptive Child Behavior Post-Intervention*

|  | ***B*** | ***SE*** | ***p*** |
| --- | --- | --- | --- |
| Condition | 0.18 | 0.35 | .60 |
| Corporal punishment | -.0.01 | 0.04 | .75 |
| Shouting | -0.02 | 0.03 | .62 |
| Praise | 0.02 | 0.03 | .62 |
| Tangible rewards | 0.01 | 0.04 | .76 |
| Monitoring | -0.01 | 0.03 | .86 |
| Threatening | 0.03 | 0.03 | .31 |
| Laxness | 0.00 | 0.03 | .98 |
| Condition-×-corporal punishment | 0.03 | 0.06 | .60 |
| Condition-×-shouting | 0.01 | 0.04 | .85 |
| Condition-×-praise | -0.05 | 0.04 | .21 |
| Condition-×-tangible rewards | 0.03 | 0.04 | .44 |
| Condition-×-monitoring | -0.02 | 0.04 | .66 |
| Condition-×-threatening | 0.01 | 0.03 | .96 |
| Condition-×-laxness | -0.08 | 0.04 | .04 |
|  |  |  |  |


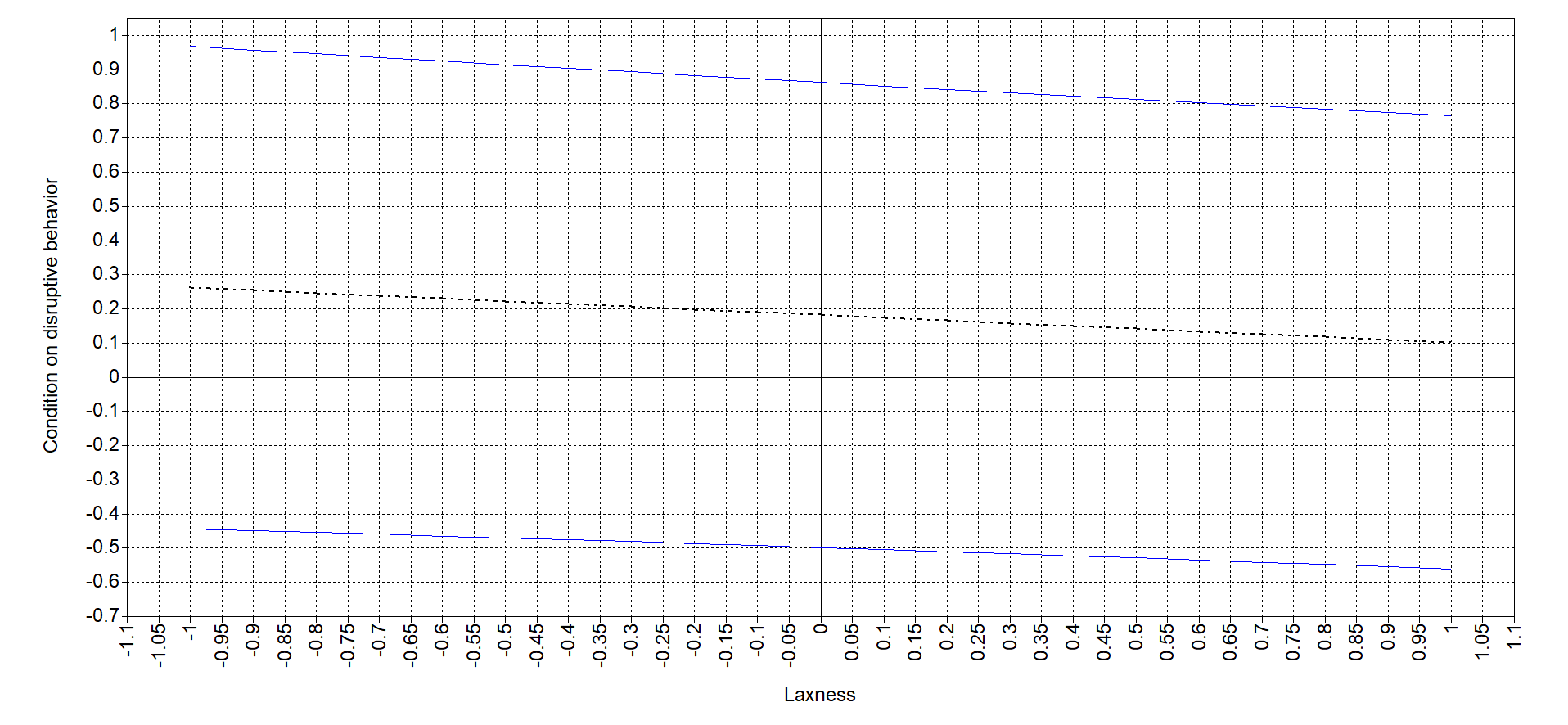


*Figure 6.1.*

Interaction Effect Between Condition and Laxness Pre-Intervention on Disruptive Child Behavior Post-Intervention

*Note.* Dashed-dotted line is estimated effect of condition on disruptive child behavior. Solid lines are upper and lower boundaries of 95% confidence intervals of that effect.
